# Scaffold Searching of FDA and EMA-Approved Drugs Identifies Lead Candidates for Drug Repurposing in Alzheimer’s Disease

**DOI:** 10.3389/fchem.2021.736509

**Published:** 2021-10-22

**Authors:** Sergey Shityakov, Ekaterina V. Skorb, Carola Y. Förster, Thomas Dandekar

**Affiliations:** ^1^ Laboratory of Chemoinformatics, Infochemistry Scientific Center, ITMO University, Saint-Petersburg, Russia; ^2^ Department of Anaesthesiology, Intensive Care, Emergency and Pain Medicine, Würzburg University Hospital, Würzburg, Germany; ^3^ Department of Bioinformatics, Biocenter, University of Würzburg, Würzburg, Germany

**Keywords:** scaffold search, approved drugs, drug repurposing, alzheimer's disease, chemical similarity, molecular modeling

## Abstract

Clinical trials of novel therapeutics for Alzheimer’s Disease (AD) have consumed a significant amount of time and resources with largely negative results. Repurposing drugs already approved by the Food and Drug Administration (FDA), European Medicines Agency (EMA), or Worldwide for another indication is a more rapid and less expensive option. Therefore, we apply the scaffold searching approach based on known amyloid-beta (Aβ) inhibitor tramiprosate to screen the DrugCentral database (*n* = 4,642) of clinically tested drugs. As a result, menadione bisulfite and camphotamide substances with protrombogenic and neurostimulation/cardioprotection effects were identified as promising Aβ inhibitors with an improved binding affinity (Δ*Gbind*) and blood-brain barrier permeation (logBB). Finally, the data was also confirmed by molecular dynamics simulations using implicit solvation, in particular as Molecular Mechanics Generalized Born Surface Area (MM-GBSA) model. Overall, the proposed *in silico* pipeline can be implemented through the early stage rational drug design to nominate some lead candidates for AD, which will be further validated *in vitro* and *in vivo*, and, finally, in a clinical trial.

## Introduction

Alzheimer’s Disease (AD) is a progressive neurodegenerative disorder, causing memory loss and in 60–70% of cases leading to dementia (Caltagirone et al., 1983). The AD pathology is widely believed to be associated with the production of β-amyloid peptide (Aβ), which is responsible for the plaque formations in the brain, disrupting normal neuronal functions (Takahashi et al., 2017). This pathological hallmark of AD might be considered in rational drug design and discovery as a promising therapeutic target to develop effective medication against this disorder. However, there is still a lack of efficient treatment for this disabling and ultimately fatal disease, e.g., donepezil and memantine usually provide at best only temporary and incomplete symptomatic relief (Nie et al., 2011). Moreover, various attempts had been made to use different drug binding sites (subregion-targets) in Aβ that could stop its aggregation and formation of a senile plague (Nie et al., 2011). In particular, tramiprosate (3-Aminopropanesulfonic acid, TRA), a mimic of glycosaminoglycans, targets the HHQK subregion at the N-terminus of Aβ (Nie et al., 2011). The TRA treatment of TgCRND8 mice resulted in a 30% reduction in the brain plaque load and the same decrease in the cerebral levels of soluble and insoluble Aβ (Gervais et al., 2007). Additionally, a dose-dependent 60% reduction of plasma Aβ levels was also observed, suggesting that this influences the Aβ central pool, changing either its efflux or its metabolism in the brain (Gervais et al., 2007). Despite the structural simplicity, high specificity, and excellent *in vivo* Aβ inhibition properties of TRA, it subsequently failed in the late stages of phase III clinical trial (Rauk, 2008). However, the data obtained from *in vitro/vivo* experiments and clinical trials could provide valuable evidence that Aβ inhibitors and their scaffolds represent a viable drug designing methodology for AD treatment (Blazer and Neubig, 2009). Indeed, some scaffold-based techniques, such as scaffold hopping, have become a powerful tool to determine the most promising drug-like candidates and already approved drugs in a drug repurposing protocol for AD (Kowal et al., 2019; Ballard et al., 2020). For example, some researchers had already performed *in silico* screening of a virtual library of a biaryl scaffold-containing compounds to inhibit the primary targets of AD therapeutics, such as acetylcholinesterase, β-secretase, monoamine oxidases, and N-methyl-D-aspartate receptor (Khalid et al., 2018). On the other hand, the scaffold searching of FDA and EMA-approved drug libraries was not previously performed to identify lead candidates for drug repurposing in AD. Moreover, the blood-brain Barrier (BBB) permeation of drug-like molecules is often considered in various pharmacokinetics (PK) studies as a pivotal PK-related descriptor (Shityakov et al., 2012; Carpenter et al., 2014). Therefore, in our study, we wanted to apply the molecular search based on the TRA scaffold to examine a database of FDA and EMA-approved drugs using *in silico* computational approaches, such as virtual screening, molecular dynamics, and BBB-based descriptor analyses.

**GRAPHICAL ABSTRACT:**
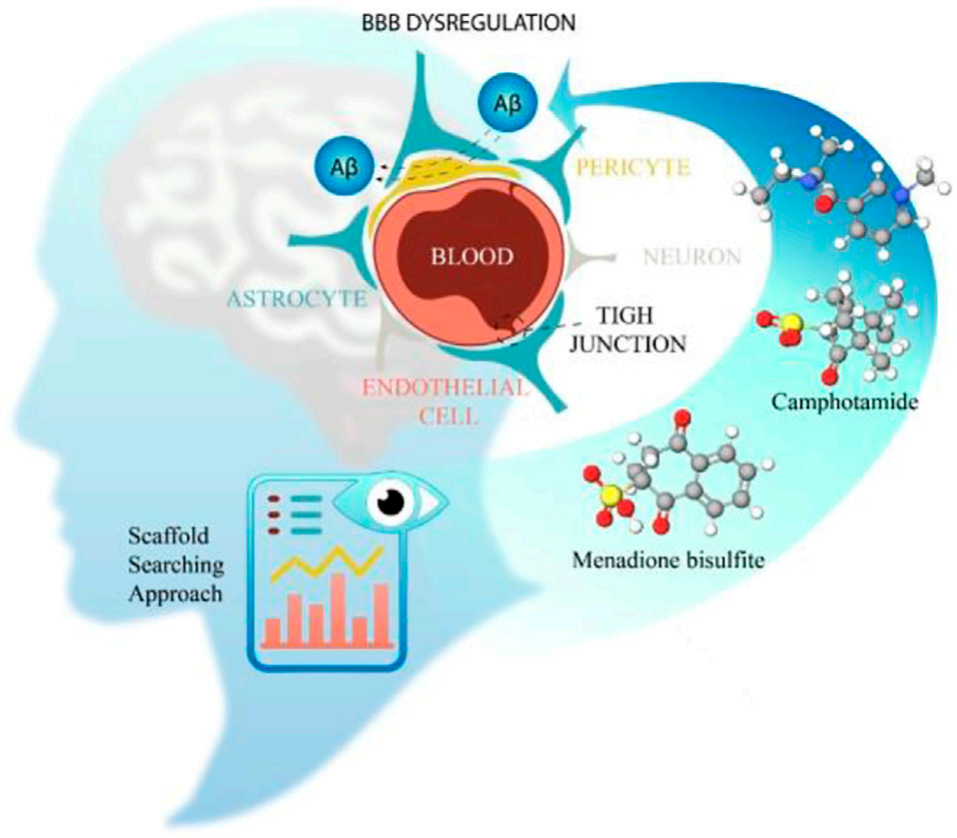


## Computational Methods

The 3D molecular structure (PBD ID: 2NAO) of a disease-relevant Aβ fibril (1–42), containing chains A, B, and C, was downloaded from the Protein Data Bank (Walti et al., 2016) to be used in the study. The Ramachandran plot server (https://zlab.umassmed.edu/bu/rama/index.pl) was implemented for the stereochemical validation of the receptor molecule to investigate the ϕ–ψ dihedral angles in a Ramachandran plot. Altogether, observed statistics showed that 86.54% (90 residues) and 11.54% (12 residues) of all observed residues were in the core and allowed regions. Additionally, no steric clashes were detected in the peptide structure. The TRA molecule and its propanesulfonic scaffold were built by using the MarvinSketch software (ChemAxon, Hungary). A database, containing the FDA and EMA-approved drugs (*n* = 4,642), was obtained from the DrugCentral 2021 online drug compendium. The Molsoft ICM 3.8-3 scaffold search algorithm was used to filter the database to identify scaffold-containing drugs. All ligands were protonated at pH = 7.4 and T = 310 K using the MOE software. Prior to molecular docking, the CASTp (Computed Atlas of Surface Topology of proteins) algorithm (Naghibzadeh, 2001) was implemented to detect the location of the peptide-ligand binding site with Cartesian coordinates located at the grid center: x = 11.87 Å; y = 18.88 Å; z = −27.75 Å. The AutoDock molecular docking algorithm to calculate binding affinity (*ΔG*_*bind*_) was implemented via the Raccoon v1.0 modeling suite to perform virtual screening. The receptor and ligand structure preparations for molecular docking included Gasteiger partial charges assignment and rotatable bonds definition according to the standard protocol published elsewhere (Shityakov et al., 2014). The inhibition constants (*Ki*) and Ligand Efficiencies (*LE*) were calculated from the binding energy values as follows:

$$\mathrm{Ki}=\exp\left[\frac{\Delta G_{\mathrm{bind}}}{\mathrm{RT}}\right]$$

$$\mathrm{LE}=\frac{\Delta G_{\mathrm{bind}}}{N_{\mathrm{atm}}}$$

where *R* (gas constant) is 1.98 cal(mol*K) ^−1^; *T* (room temperature) is 298.15 K; *N*_*atm*_ is the number of non-hydrogen atoms in a molecule. AutoDock v.4.2.5.1 was used in the study since its previous version incorrectly calculates part of the intermolecular desolvation energy term (Goodsell et al., 1996). The docking grid with dimension size of 60 × 60 × 60 Å and a grid spacing of 0.375 Å were used in the study. The Glide molecular docking algorithm with the Prime MM-GBSA (generalized Born solvent-accessible surface area) approach to calculate the free energy of binding (*ΔG*_*PR*_) was implemented using the default settings, such as the OPLS3e force field, 0.25 of charge cutoff, and 0.8 scaling of the vdW radii of non-polar receptor and ligand atoms. The G-score value, as an empirical scoring function, was used to approximate the ligand binding free energy. All molecular dynamics (MD) simulations were performed using the AMBER 16 package with the FF99SB and GAFF force fields for the Aβ peptide and its ligands (Case et al., 2005). The Antechamber module of AmberTools was employed to calculate the partial charges of the ligands using the semi-empirical AM1-BCC function according to the standard protocol (Marques et al., 2021). The systems were solvated with the TIP3P water models and neutralized by adding the Na + ions using the tLEap input script available from the AmberTools package. Long-range electrostatic interactions were modeled via the particle-mesh Ewald method (Essmann et al., 1995). The SHAKE algorithm (Miyamoto and Kollman, 1992) was applied to constrain the length of covalent bonds, including the hydrogen atoms. Langevin thermostat was implemented to equilibrate the temperature of the system at 310 K. A 2.0-fs time step was used in all of the MD setups. For the minimization and equilibration (NVT and NPT ensembles) phases, 100,000 steps and a 1-ns period were used, respectively. Finally, 100-ns classical MD simulations, with no constraints as NPT ensemble, were performed for each of the peptide-ligand complexes using the molecular mechanics combined with the Poisson–Boltzmann (MM-PBSA) or generalized Born (MM-GBSA) augmented with the hydrophobic solvent-accessible surface area term (Kollman et al., 2000; Shityakov et al., 2017). The MM-PBSA/GBSA solvation models were applied as a post-processing end-state method to calculate the free energies (*ΔG*_*PB*_ and *ΔG*_*GB*_) together with the entropies (*TΔS*) and enthalpies (*ΔH*) for the analyzed molecules, namely:

$$\Delta G_{\mathrm{PB}}\;=\Delta H_{\mathrm{PB}}–\mathrm{T\Delta S};\Delta G_{\mathrm{GB}}\;=\Delta H_{\mathrm{GB}}–\mathrm{T\Delta S}$$

To calculate the blood-brain barrier partitioning coefficients (*logBB*), the in-house python script, based on the Clark (*logBB*_*cl*_) and Rishton (*logBB*_*ri*_) linear regression models, was ran according to the following equations (Clark, 2003; Rishton et al., 2006):

$$\mathrm{logB}B_{\mathrm{cl}}=0.152\mathrm{AlogP}-0.0148\mathrm{PSA}+0.139$$

$$\mathrm{logB}B_{\mathrm{ri}}=0.155\mathrm{AlogP}-0.01\mathrm{PSA}+0.164$$

where *PSA* and *AlogP* are the polar surface area and atom-based octanol-water partitioning coefficient. The in-house PyMol script was applied to calculate the Buried Surface Area (*BSA*) of the peptide-ligand complexes according to the equation:

$$\mathrm{BSA}=\frac{\left(\mathrm{AS}A_{\mathrm{pep}}+\mathrm{AS}A_{\mathrm{lig}}\right)-\mathrm{AS}A_{\mathrm{comp}}}{2}$$

where *ASA*_*pep*_ and *ASA*_*lig*_ and *ASA*_*comp*_ are the accessible surface areas of the peptide, ligand, and complex components. Molecular descriptors, such as molecular weight (*MW*), *AlogP*, H-bond acceptors (*HBA*), H-bond donors (*HBD*), *PSA*, quantitative estimate of drug-likeness (*QED*), and Tanimoto molecular similarity (*T*) indexes were calculated with the Biscu-it™ tools and Rcpi and Rcdk libraries within the *Python* and R environments (Voicu et al., 2020; Cao et al., 2015). Buried surface areas (*BSA*) have been calculated for 2D Aβ-ligand interaction diagrams using the NACCESS program (Hubbard and Thornton, 1993; Wallace et al., 1995). Molecular graphics and visualization were performed with the LigPlot + program (EMBL-EBI, Wellcome Trust Genome Campus, Hinxton, United Kingdom) in order to build two-dimensional interaction diagrams from three-dimensional coordinates. Linear regression analysis, followed by graphical representation was performed by PyMol and GraphPad Prism v.8 (GraphPad Software, San Diego, CA, United States). The differences were considered statistically significant at a *p*-value of <0.05.

## Results and Discussion

Before molecular docking and MD simulations, the DrugCentral database (n = 4,642) was filtered to find the molecules, containing the propanesulfonic scaffold of TRA. The scaffold search protocol is based on the chemical similarity searching that can be used to screen a database of compounds for structural similarity to a query chemical structure. The small portion (*n* = 13) of FDA and EMA-approved drugs (Table 1), containing propanesulfonic scaffold of TRA, was identified as several hit molecules suitable for further investigation using molecular docking to eventually locate some lead candidates. In fact, our study relies on the modified hit-to-lead protocol to identify promising lead compounds, inhibiting Aβ in order to highlight the possibility for their optimization.

**TABLE 1:** Chemical structures and clinical indications of FDA and EMA-approved drugs (*n* = 13) containing propanesulfonic scaffold of TRA.

Compound	Structure	Indication
1. Unithiol	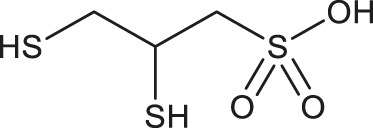	Mercury, arsenic, and lead poisoning
2. Aurotioprol	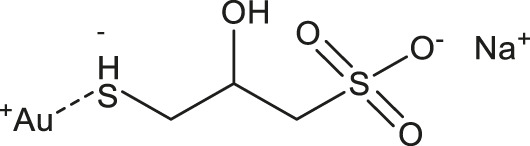	Moderate-severe rheumatoid arthritis and tuberculosis
3. Acamprosate	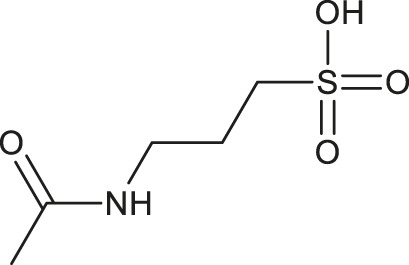	Abstinence from alcohol
4. Menadione	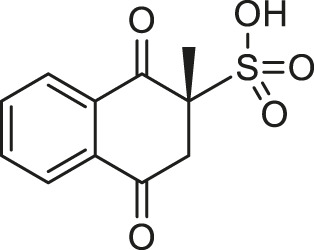	Hypoprothrombinemia
5. Docusate sodium	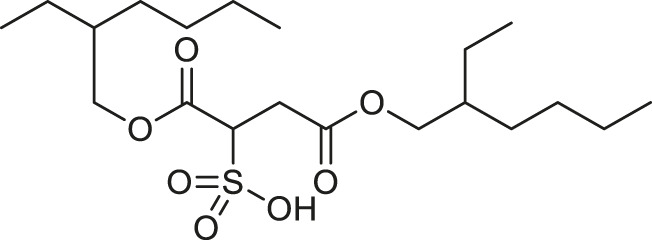	Occasional constipation
6. Camphotamide	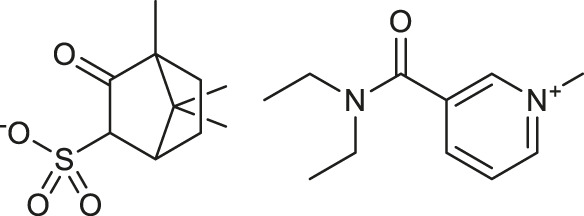	Cardioprotection and neurostimulation
7. Dermatan sulfate	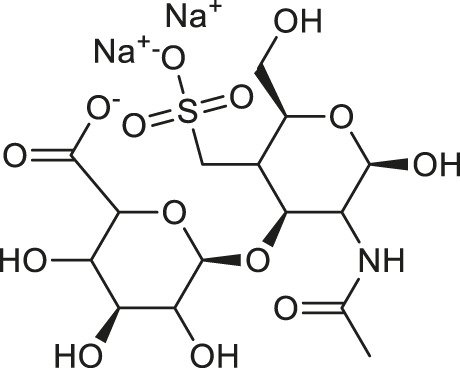	Deep vein thrombosis
8. Ecamsule	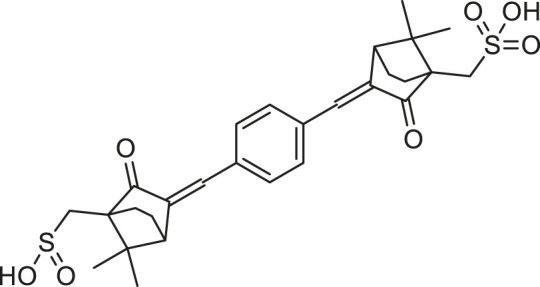	Skin protection
9. Sulfamazone	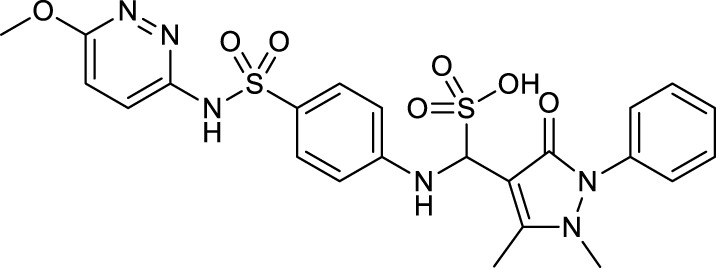	Sulfonamide antibiotic with antipyretic properties
10. Cefpimizole	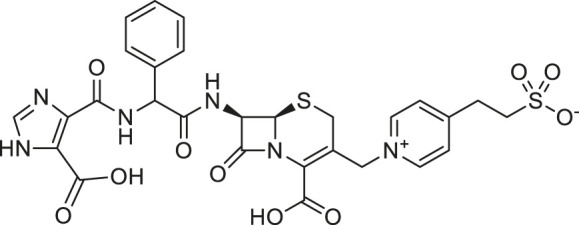	Infections of skin or soft tissue and urinary tract
11. Glucosulfone	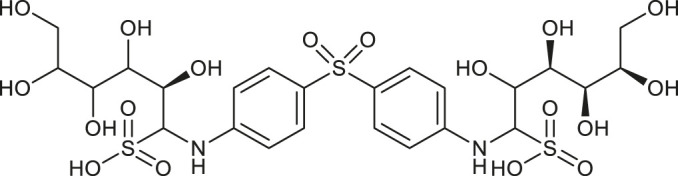	Treatment of malaria tuberculosis and leprosy
12. Solasulfone	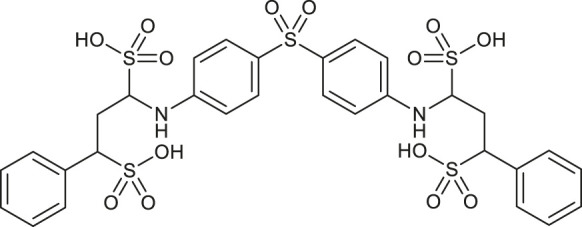	Treatment of leprosy
13. Indocyanine green	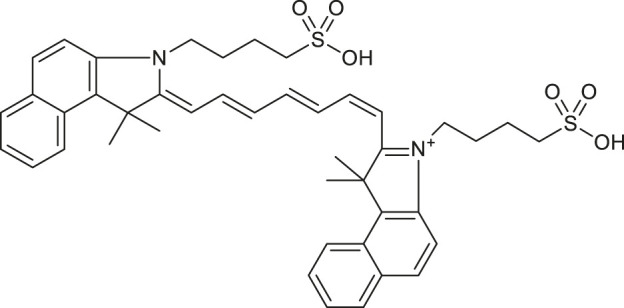	Ophthalmic angiography and treatment of cancer and acne vulgaris
14. TRA [scaffold]	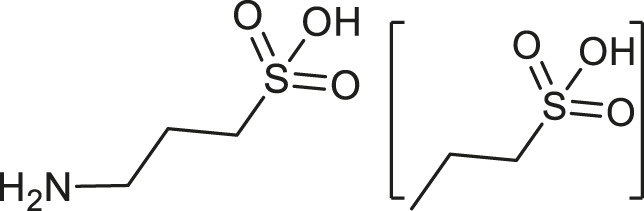	AD (failed in phase III)

The identified hit molecules belong to different chemical classes and can be prescribed to treat various pathological conditions, such as bacterial infections (leprosy and tuberculosis), lead or mercury poisoning, metabolic disorders, and neurological conditions. Subsequently, the rigid-flexible molecular docking was performed to calculate the affinity between hit substances and Aβ, targeting the HHQK subregion at the N-terminus of the peptide. The lowest-energy Aβ conformer of the NMR models (*n* = 10) was chosen as a receptor molecule for molecular docking with the potential energy of -1,106.70 kcal/mol, which makes it more stable in the solution. Additionally, the 3D alignment of the Aβ peptide and HHQK region reviled the 2.0 Å structural deviation indicating a relatively “conserved” protein-receptor binding site in solution NMR models (Supplementary Figures S1A,B).

**FIGURE 1:**
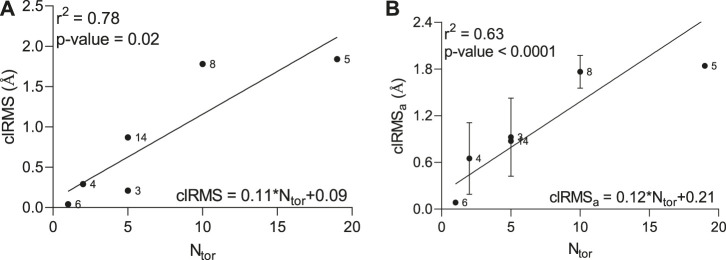
Relationship between clRMS **(A)** as a root-mean-square difference between top two conformations in the lowest-energy cluster, clRMSa **(B)** as an average of the root-mean-square difference between all conformations and the lowest energy conformation in the lowest-energy cluster and the number of torsions (*N*_*tor*_) in the ligand molecule.

To accurately assess any correlations between the number of atoms in the ligand molecule and the conformational effects happening within the peptide-ligand binding site, we estimated the root-mean-square (*RMS*) difference between the top two conformations and its average value calculated between all conformations and the lowest-energy conformation in the lowest-energy cluster (Supplementary Figure S2). A strong positive correlation (*r* ^2^ = 0.78) with reliable statistics (*p*-value = 0.02) between the *clRMS*, as an *RMS* difference between top two conformations in the largest cluster, and the number of torsions (*N*_*tor*_) was observed by linear regression analysis (Figure 1A). Furthermore, a significant standard deviation of the *clRMSa* variable, as an average of the *RMS* difference between all conformations and the lowest energy conformation, was detected for some hit compounds (3, 4, 8, and 14), describing the elevated conformational change (Figure 1B), which greatly affects the correlation coefficient (*r* ^2^ = 0.63).

**FIGURE 2:**
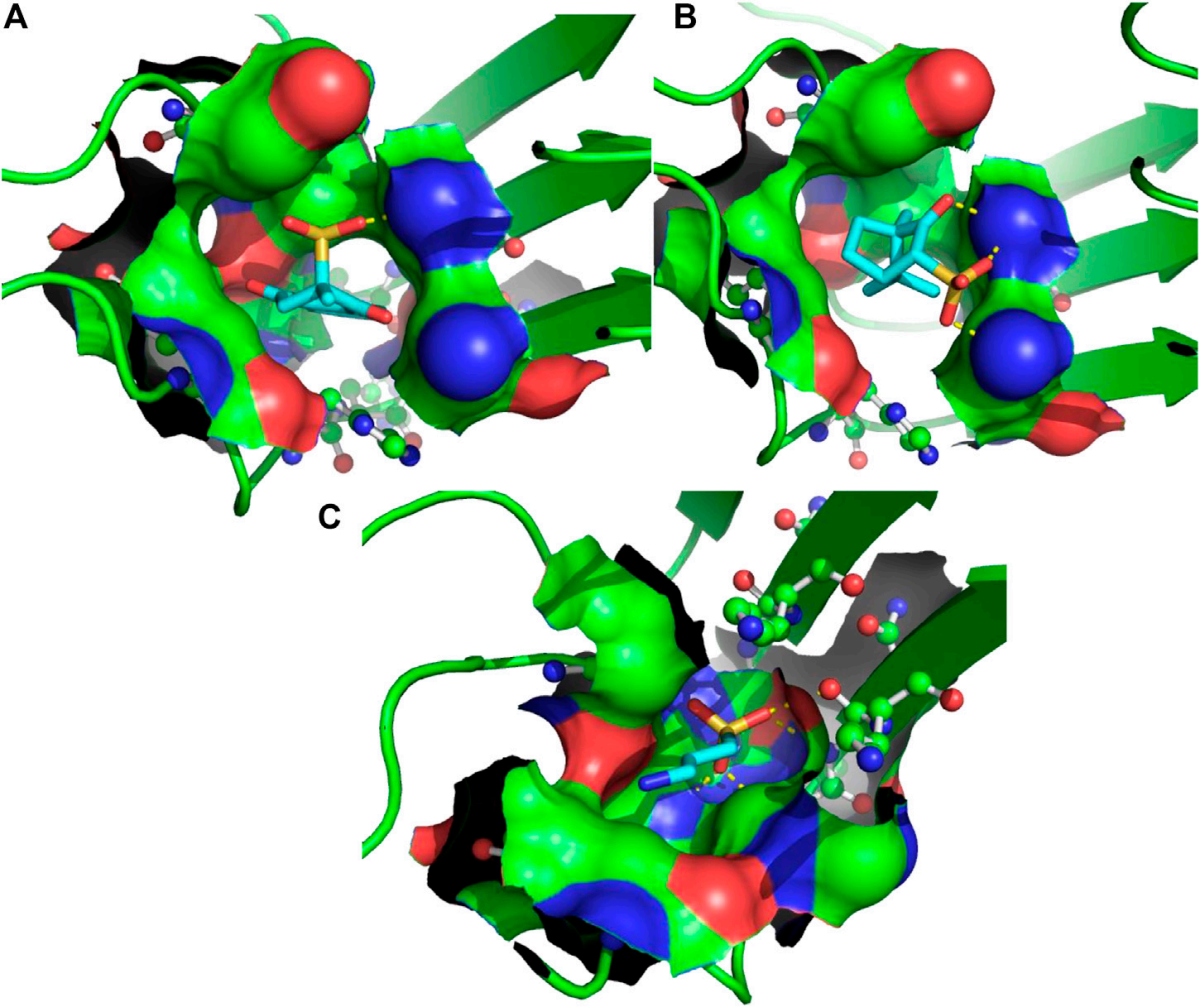
3D binding modes predicted from the AutoDock runs for menadione **(A)**, camphotamide **(B)**, and TRA **(C)** bound to Aβ peptide. The peptide-ligand binding site is shown by the molecular surface and colored according to the peptide atomic composition. The peptide is shown as a ribbon diagram, and its residues are drawn as ball-and-stick models. Hydrogen bonds are visualized as a dashed lines. The ligand molecules are depicted in sticks, and hydrogen atoms are removed to enhance clarity.

Finally, menadione and camphotamide lead candidates were found among the top binders (Table 2 and Figures 2A,B) with the binding energy values (−7.11 and −6.82 kcal/mol) significantly lower than for the reference compound with a *ΔG*_*bind*_ value of −6.19 kcal/mol (Table 2 and Figure 2C). It was previously published that menadione sodium bisulfite as a remedy against hemorrhagic disease caused by vitamin K deficiency could inhibit Aβ toxic formation and aggregation in *C. elegans*, extending its life span and reducing disruption of cellular membranes (Zhang et al., 2018). The co-authors also hypothesized that menadione might inhibit amyloid formation due to its backbone similarity to 1,4-naphthoquinone, which shows strong anti-aggregation effects on amyloidogenic proteins, such as insulin and α-synuclein. Similarly, the 6-hydroxy-nicotine intermediate from *P. nicotinovorans* as a precursor for camphotamide has been shown to have neuroprotective effects with putative applications in AD treatment (Wang et al., 2015; Hritcu et al., 2017). As a positive inotropic agent (cardiotonic), this substance could be potentially prescribed to treat AD-associated amyloid cardiomyopathy, inhibiting, directly and indirectly, Aβ accumulation in the heart. Indeed, the inhibition of Aβ and the formation of neurofibrillary tangles along the heart-brain axis could be reached through targeted supplementation of neurotrophic factors to the brain as it was hypothesized by Shityakov and coauthors Shityakov et al. (2021).

**TABLE 2:** Summary of AutoDock molecular docking results for drugs (n = 13), containing propanesulfonic scaffold of TRA.

Compound	ΔG_bind_ kcal/mol	clRMSD Å	clRMSD_a_ Å	N_atm_	N_tor_	Ki µM	LE	T
1	−3.05	—	—	12	6	5.7^a^	−0.25	0.57
2	−3.7	—	—	11	5	1.89^a^	−0.34	0.67
3	−5.15	0.21	0.74	13	5	162.66	-0.39	0.71
4	−7.11	0.29	0.54	18	2	5.87	−0.39	0.56
5	−4.97	1.84	1.84	29	19	220.65	-0.17	0.63
6	−6.82	0.04	0.07	15	1	9.61	−0.46	0.5
7	−4.26	—	—	37	12	734.58	−0.12	0.56
8	−6.17	1.78	1.18	40	10	28.9	−0.15	0.45
9	−5.91	—	—	41	10	44.89	−0.14	0.33
10	−2.97	—	—	52	15	6.53^a^	−0.06	0.27
11	0.66	—	—	61	30	3.06^b^	0.01	0.44
12	−3.81	—	—	57	20	1.57^a^	−0.07	0.44
13	−5.71	—	—	55	16	62.99	−0.1	0.42
14	−6.19	0.87	0.58	11	5	27.94	−0.56	0.67

a mM.

b M.

**FIGURE 3:**
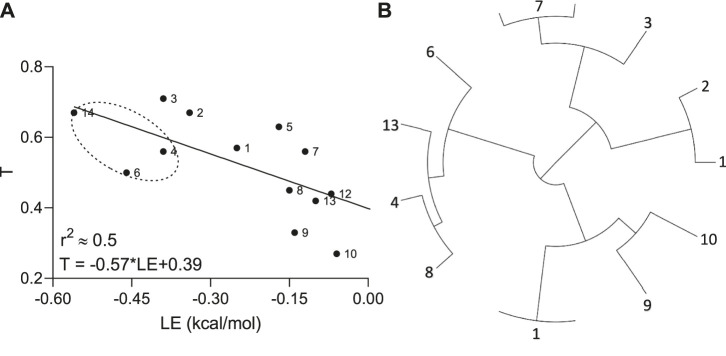
Relationship **(A)** between Tanimoto similarity (T) and ligand efficiency (LE) descriptors and tree visualization **(B)** of the hierarchical clustering based on structural similarities calculated for hit molecules (*n* = 13), containing propanesulfonic scaffold of TRA.

To investigate how molecular similarity contributes to the Aβ binding, the Tanimoto coefficients as an appropriate choice for a fingerprint-based similarity and ligand efficiency as the affinity normalized by the number of non-hydrogen atoms were implemented. A moderate negative correlation (*r* ^2^ ≈ 0.5) with reliable statistics (*p*-value = 0.008) between the *T* and *LE* parameters was observed by linear regression analysis (Figure 3).

In other words, if chemical compounds match more to the TRA scaffold, they also have very similar binding modes calculated per number of heavy atoms in the molecule. In addition, all hit molecules were subdivided into 4 big groups or clusters by the hierarchical clustering according to their E-state molecular fingerprints and pairwise similarity matrix, where the lead molecules (compounds 4 and 6) ended up in the same cluster (Figure 3B).

Next, the *AlogP*, *QED*, *PSA*, and *logBB* values for menadione, camphotamide, and TRA as a reference, and donepezil as standard control were calculated to evaluate the drug ability to possess the optimal BBB permeation properties and druglikness (Table 3).

**TABLE 3:** Summary of molecular descriptors and partitioning coefficients (*logBB*) determined for lead compounds (drugs) with highest Aβ inhibition properties.

Compound	MW	AlogP	HBA	HBD	PSA	QED	logBB_cl_	logBB_ri_	logBB_exp_
Menadione	253.25	0.98	4	0	88.18	0.76	−1.02	−0.57	—
Camphotamide	231.29	1.14	3	0	71.11	0.69	−0.74	−0.37	—
TRA	138.17	−0.91	3	1	80.06	0.58	−1.18	−0.78	—
Donepezil	379.5	4.36	4	0	38.77	0.72	0.23	0.45	0.89

The *AlogP* parameter for lead candidates suggested their mild lipophilic properties, which were significantly higher than for the reference substance but not as good as for the standard control. On the other hand, the *QED* parameters for lead candidates were very similar to those determined for donepezil, reflecting the distribution of molecular properties, such as the number of hydrogen bond donors and acceptors, the number of aromatic rings, and the presence of unwanted chemical functionalities. However, for a CNS-active compound to permeate the BBB, a surface area less than 60–70 Å^2^ is usually required to achieve the desired brain bioavailability for the administered drug (Shityakov et al., 2014; Kelder et al., 1999). Judging by relatively low *AlogP* and high *PSA*, there is a risk that the BBB permeation of the lead candidates might not be entirely sufficient as confirmed by the logBB, which optimally should be *logBB* > 0 (Shityakov et al., 2015). Therefore, further lead optimization by *in situ* enumeration or fragment replacement might be a plausible choice to make chemical modifications, improving brain bioavailability (*logBB*) together with selectivity, pharmacokinetic and pharmacodynamic parameters, and decreasing toxicity. Reaction-based *in situ* enumeration can be achieved by proposing synthetically feasible candidates from a reagent library in particular, which reagents are most likely to produce potential active compounds for the binding site (Mok et al., 2017). On the contrary, the fragment replacement protocol performs scaffold hopping by replacing part of the scaffold structure while maintaining the favorable binding between the receptor and the ligand (Vainio et al., 2013). The elevated experimental *logBB* index of 0.89 for donepezil was mainly due to its carrier-mediated transport to the brain (Kim et al., 2010). It could probably be accomplished by organic cation or choline transporters (OCT1-3 and CHT1) and not just crossing the BBB via passive diffusion through endothelial cells (Kim et al., 2010).

To validate further molecular docking results, the free energy of binding based on implicit solvation models was calculated for amyloid-drug complexes. The MM-PBSA/GBSA calculations (Table 4), using 100 ns MD trajectories, confirmed the previous data completely (GBSA) and partially (PBSA), revealing much higher binding affinities of lead compounds to Aβ in comparison to the reference.

**TABLE 4:** Summary of binding affinities (ΔG and G-score), entropy (TΔS), enthalpy (ΔH_PB_ and ΔH_GB_), and Buried Surface Area (BSA) values calculated for lead candidates and TRA. Entropy-enthalpy compensation data of the peptide-ligand complexes is obtained from the normal-mode analyses of 100 ns trajectories at the temperature of 298.15 K.

Compound	ΔG_PB_	ΔG_GB_	ΔG_PR_	G-score kcal/mol	TΔS	ΔH_PB_	ΔH_GB_	BSA, Å^2^
Menadione	−13.75	−19.71	−38.81	−6.51	−20.4	−34.15	−40.11	489.77
Camphotamide	−15.61	−14.86	−21.36	−5.08	−17.86	−33.47	−32.72	481.09
TRA	−2.28	−10.1	−16.31	−4.49	−17.91	−20.19	−28.01	373.56

**FIGURE 4:**
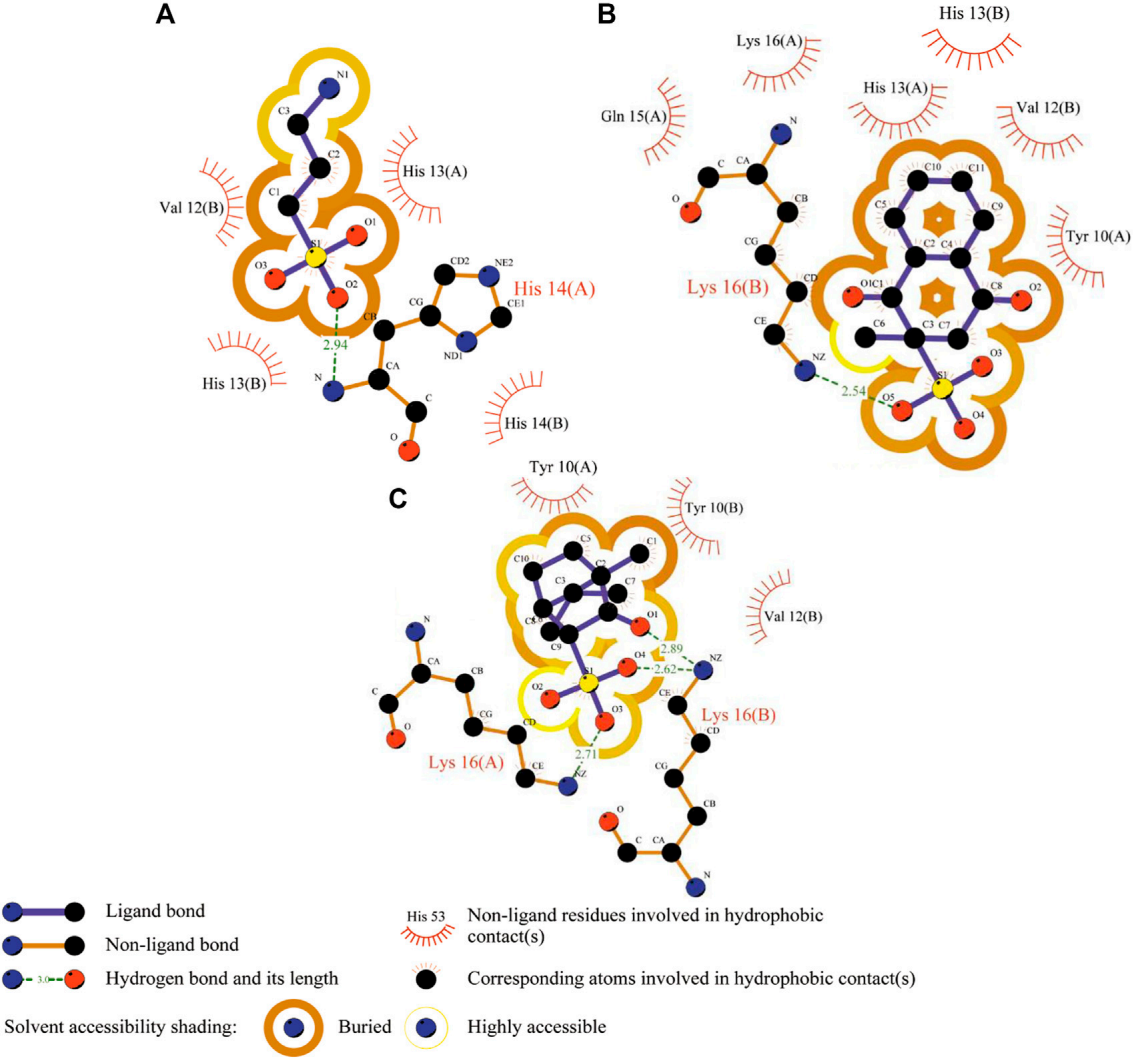
2D Aβ-ligand interaction diagrams predicted from the AutoDock runs for TRA **(A)**, menadione **(B)**, and camphotamide **(C)** bound to Aβ peptide. The chain information is shown in parentheses. All hydrogen atoms are removed to enhance clarity.

The former protocol provided the best affinity for menadione (*ΔG*_*GB*_ = −19.71 kcal/mol), probably because in some extensive studies the MM-GBSA approach was computationally more efficient, achieving better accuracy but being less rigorous (Hou et al., 2011). Therefore, the second validation was done to clarify the MM-PBSA/GBSA discrepancy by utilizing the Prime algorithm with the MM-GBSA-based scoring function. As a result, the G-score data and the entropy-enthalpy compensation analysis confirmed the previous findings, describing the exothermic nature (*ΔH* < 0) of the binding process with the decreased disorder (*TΔS* <0), which could occur spontaneously depending on temperature. Additionally, the *BSA* values (Table 4 and Figures 4A–C), which measures the size of the Aβ-drug interface, also confirmed the previous binding affinity pattern, where the *BSA* elevation leads to an increase in binding as it was already published for the peptide-ligand and host-guest systems (Shityakov et al., 2020; Esmaeilpour et al., 2021).

To analyse the movements of the studied complexes, the root-mean-square deviation (*RMSD*) and fluctuation (*RMSF*) values together with the radius of gyration (*R*_*g*_) with respect to the initial conformation were plotted versus time (Supplementary Figures S3A–E). The receptor *RMSD* values were relatively high (*RMSD* = 10 Å) stabilized after about 20 ns for the compound 4 and 6/Aβ complexes and after 40 ns for TRA/Aβ (Supplementary Figure S3A). The ligand *RMSD* remained low within 1.0 Å and stabilized almost instantly (Supplementary Figure S3B). The receptor *RMSF* values produced some picks associated with the high flexibility of the Aβ termini (Supplementary Figure S3C). The atomic fluctuations of ligands showed more stable profiles especially for compounds 4 and 6 (Supplementary Figure S3D). The decreased *R*_*g*_ values were associated with the receptor compactness, which was elevated during the simulation (Supplementary Figure S3E). Finally, the number of H-bonds between the receptor and its ligands and the fraction of residues involved in H-bonding were assessed to find the H-binding contribution to the affinity. These parameters were the highest for compound 4 with more H-bonds formed, (Supplementary Figure S3F) significantly increasing the residue fraction (Supplementary Figure S3G).

**FIGURE 5:**
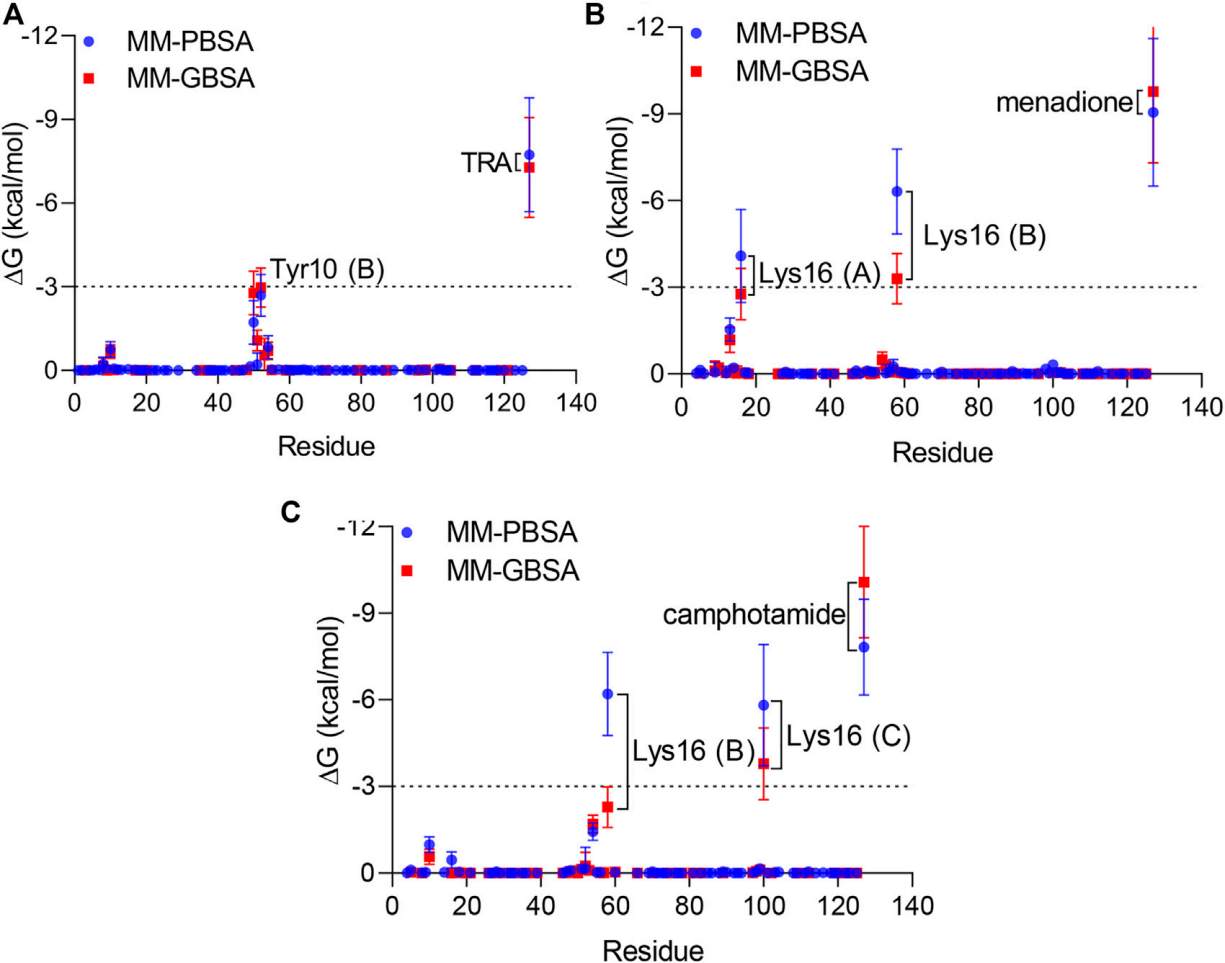
Per-residue energy decomposition analysis using 100 ns MD trajectories of TRA **(A)**, menadione **(B)**, and camphotamide **(C)** bound to Aβ peptide as implicit solvation MM-PBSA/GBSA models. The energy threshold is depicted as dashed line. The information about Aβ chains is added in parentheses.

**FIGURE 6:**
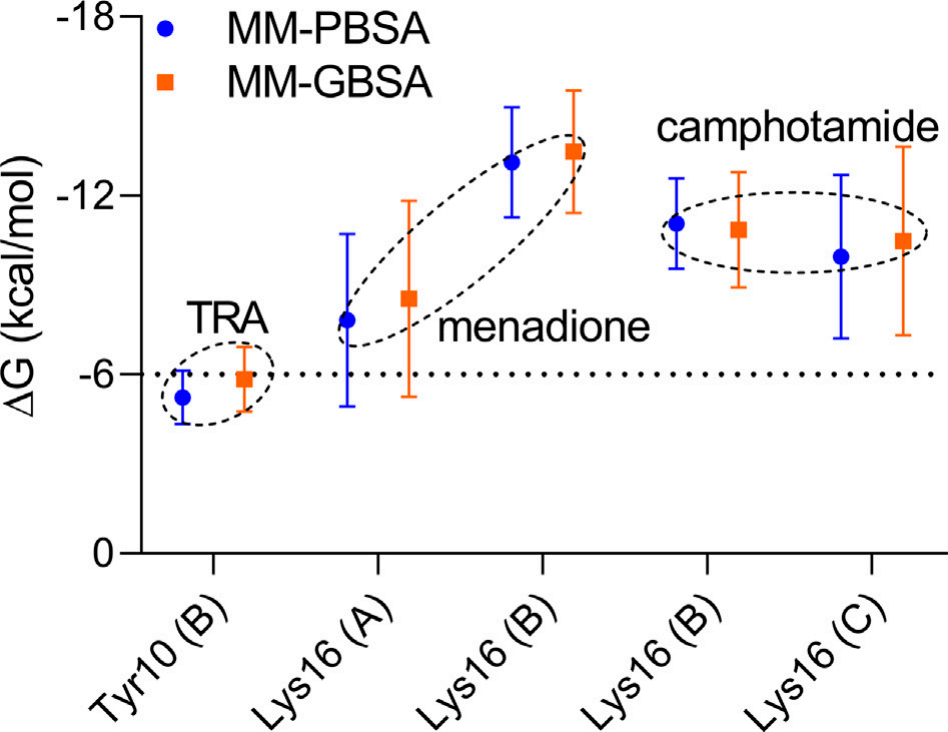
Pairwise energy decomposition analysis using 100 ns MD trajectories of analyzed compounds bound to Aβ peptide as implicit solvation MM-PBSA/GBSA models. The information about Aβ chains is added in brackets. The energy threshold is depicted as a dotted line.

Finally, per-residue and pairwise energy decomposition analyses were employed to evaluate the energetic contribution of drugs and the binding site residues. Some unique residues, such as Tyr10 (B) for TRA, Lys16 (A, B) for menadione, and Lys16 (B, C) for camphotamide were identified at the energetic threshold (*ΔG* = −3.0 kcal/mol) and below by one or both implicit solvation protocols (Figures 5, 6). In particular, all amino acid residues involved in the interaction with the lead candidates had exceeded the energetic threshold (Figure 5A,B), which was not observed for the reference molecule (Figure 5C). Meanwhile, the Lys16 targeting in Aβ by the oxidation of a catechol structure to the *o*-quinone forming the *o*-quinone-Aβ adduct is believed to be responsible for its anti-aggregation activity (Liu et al., 2017).

In the pairwise interaction, only Tyr10 (B) was detected slightly above the adjusted energetic threshold (*ΔG* = −6.0 kcal/mol), and Lys16 (B) had the lowest energy during the MD simulation of the Aβ-menadione complex (Figure 6). Highlighting the contribution of these residues for the inhibitor of this site, curcumin and resveratrol were found to interact with Arg5, Ser8, Tyr10, Gln15, Lys16, Leu17, and Phe20 (Fu et al., 2014). Besides, solvent-accessible residues, such as Phe20 at the N-terminal.

KLVFF stretch was reported as crucial in the interactions of these two inhibitors (Fu et al., 2014). Some studies have also documented the pairwise interactions for some amyloid variants (Aβ_10-35_) to illustrate residue energetic contributions in a process of Aβ reorganization driven basically by inter-chain hydrophobic and hydrophilic interactions and also solvation/desolvation effects (Campanera and Pouplana 2010).

## Conclusion

In this study, the scaffold searching approach based on known Aβ inhibitor tramiprosate to screen the DrugCentral database (*n* = 4,642) was employed to identify hit compounds (*n* = 13) and lead candidates (*n* = 2) for AD drug repurposing. Two lead candidates, namely menadione bisulfite and camphotamide, out of 13 hit compounds were identified as promising Aβ inhibitors with the improved *ΔG*_*bind*_ and *logBB* parameters. The binding affinity modes were also confirmed by molecular dynamics simulations using implicit solvation models, in particular MM-GBSA. We assume that this scaffold searching methodology in conjunction with pharmaceutical profiles (*logBB*) can be applied as a starting routine for drug repurposing in AD. Overall, the proposed computational pipeline can be implemented through the early stage rational drug design to nominate drugs for AD that, after additional *in vitro* and *in vivo* validation, could be readily evaluated in a clinical trial.

## Data Availability

The original contributions presented in the study are included in the article/Supplementary Material, further inquiries can be directed to the corresponding authors.

## References

[B1] Ballard C. Aarsland D. Cummings J. O'Brien J. Mills R. Molinuevo J. L. (2020). Drug Repositioning and Repurposing for Alzheimer Disease. Nat. Rev. Neurol. 16 (12), 661–673. 10.1038/s41582-020-0397-4 32939050 PMC8291993

[B2] Blazer L. L. Neubig R. R. (2009). Small Molecule Protein-Protein Interaction Inhibitors as CNS Therapeutic Agents: Current Progress and Future Hurdles. Neuropsychopharmacology 34 (1), 126–141. 10.1038/npp.2008.151 18800065

[B3] Caltagirone C. Albanese A. Gainotti G. Masullo C. (1983). Acute Administration of Individual Optimal Dose of Physostigmine Fails to Improve Mnesic Performances in Alzheimers Presenile Dementia. Int. J. Neurosci. 18 (1-2), 143–147. 10.3109/00207458308985888 6840979

[B4] Campanera J. M. Pouplana R. (2010). MMPBSA Decomposition of the Binding Energy throughout a Molecular Dynamics Simulation of Amyloid-Beta (Abeta(10-35)) Aggregation. Molecules (Basel, Switzerland) 15 (4), 2730–2748. 10.3390/molecules15042730 PMC6257327 20428075

[B5] Cao D.-S. Xiao N. Xu Q. S. Chen A. F. (2015). Rcpi: R/Bioconductor Package to Generate Various Descriptors of Proteins, Compounds and Their Interactions. Bioinformatics (Oxford, England) 31 (2), 279–281. 10.1093/bioinformatics/btu624 25246429

[B6] Carpenter T. S. Kirshner D. A. Lau E. Y. Wong S. E. Nilmeier J. P. Lightstone F. C. (2014). A Method to Predict Blood-Brain Barrier Permeability of Drug-like Compounds Using Molecular Dynamics Simulations. Biophysical J. 107 (3), 630–641. 10.1016/j.bpj.2014.06.024 PMC4129472 25099802

[B7] Case D. A. Cheatham T. E. Darden T. Gohlke H. Luo R. Merz K. M. (2005). The Amber Biomolecular Simulation Programs. J. Comput. Chem. 26, 1668–1688. 10.1002/jcc.20290 16200636 PMC1989667

[B8] Clark D. E. (2003). In Silico prediction of Blood-Brain Barrier Permeation. Drug Discov. Today 8 (20), 927–933. 10.1016/s1359-6446(03)02827-7 14554156

[B9] Esmaeilpour D. Shityakov S. Tamaddon A. M. Bordbar A. K. (2021). Comparative Chemical Examination of Inclusion Complexes Formed with Beta-Cyclodextrin Derivatives and Basic Amino Acids. Carbohydr. Polym. 262, 117868. 10.1016/j.carbpol.2021.117868 33838791

[B10] Essmann U. Perera L. Berkowitz M. L. Darden T. Lee H. Pedersen L. G. (1995). A Smooth Particle Mesh Ewald Method. J. Chem. Phys. 103, 8577–8593. 10.1063/1.470117

[B11] Fu Z. Aucoin D. Ahmed M. Ziliox M. Van Nostrand W. E. Smith S. O. (2014). Capping of Abeta42 Oligomers by Small Molecule Inhibitors. Biochemistry 53 (50), 7893–7903. 10.1021/bi500910b 25422864 PMC4278677

[B12] Gervais F. Paquette J. Morissette C. Krzywkowski P. Yu M. Azzi M. (2007). Targeting Soluble Abeta Peptide with Tramiprosate for the Treatment of Brain Amyloidosis. Neurobiol. Aging 28 (4), 537–547. 10.1016/j.neurobiolaging.2006.02.015 16675063

[B13] Goodsell D. S. Morris G. M. Olson A. J. (1996). Automated Docking of Flexible Ligands: Applications of AutoDock. J. Mol. recognition: JMR 9 (1), 1–5. 10.1002/(sici)1099-1352(199601)9:1<1:aid-jmr241>3.0.co;2-6 8723313

[B14] Hou T. Wang J. Li Y. Wang W. (2011). Assessing the Performance of the MM/PBSA and MM/GBSA Methods. 1. The Accuracy of Binding Free Energy Calculations Based on Molecular Dynamics Simulations. J. Chem. Inf. Model. 51 (1), 69–82. 10.1021/ci100275a 21117705 PMC3029230

[B15] Hritcu L. Ionita R. Motei D. E. Babii C. Stefan M. Mihasan M. (2017). Nicotine versus 6-Hydroxy-L-Nicotine against Chlorisondamine Induced Memory Impairment and Oxidative Stress in the Rat hippocampus. Biomed. Pharmacother. 86, 102–108. 10.1016/j.biopha.2016.12.008 27951416

[B16] Hubbard S. J. Thornton J. M. (1993). NACCESS. London: Department of Biochemistry and Molecular Biology, University College.

[B17] Kelder J. Grootenhuis P. D. Bayada D. M. Delbressine L. P. Ploemen J. P. (1999). Polar Molecular Surface as a Dominating Determinant for Oral Absorption and Brain Penetration of Drugs. Pharm. Res. 16 (10), 1514–1519. 10.1023/a:1015040217741 10554091

[B18] Khalid S. Zahid M. A. Ali H. Kim Y. S. Khan S. (2018). Biaryl Scaffold-Focused Virtual Screening for Anti-aggregatory and Neuroprotective Effects in Alzheimer's Disease. BMC Neurosci. 19 (1), 74. 10.1186/s12868-018-0472-6 30424732 PMC6234579

[B19] Kim M.-H. Maeng H. J. Yu K. H. Lee K. R. Tsuruo T. Kim D. D. (2010). Evidence of Carrier-Mediated Transport in the Penetration of Donepezil into the Rat Brain. J. Pharm. Sci. 99 (3), 1548–1566. 10.1002/jps.21895 19691109

[B20] Kollman P. A. Massova I. Reyes C. Kuhn B. Huo S. Chong L. (2000). Calculating Structures and Free Energies of Complex Molecules: Combining Molecular Mechanics and Continuum Models. Acc. Chem. Res. 33, 889–897. 10.1021/ar000033j 11123888

[B21] Kowal N. M. Indurthi D. C. Ahring P. K. Chebib M. Olafsdottir E. S. Balle T. (2019). Novel Approach for the Search for Chemical Scaffolds with Activity at Both Acetylcholinesterase and the Alpha 7 Nicotinic Acetylcholine Receptor: A Perspective on Scaffolds with Dual Activity for the Treatment of Neurodegenerative Disorders. Molecules 24 (3), 446. 10.3390/molecules24030446 PMC6384821 30691196

[B22] Liu M. Wan L. Bin Y. Xiang J. (2017). Role of Norepinephrine in Abeta-Related Neurotoxicity: Dual Interactions with Tyr10 and SNK(26-28) of Abeta. Acta Biochim. Biophys. Sinica 49 (2), 170–178. 10.1093/abbs/gmw126 28069584

[B23] Marques S. M. Šupolíková L. Molčanová L. Šmejkal K. Bednar D. Slaninová I. (2021). Screening of Natural Compounds as P-Glycoprotein Inhibitors against Multidrug Resistance. Biomedicines 9 (4), 357. 10.3390/biomedicines9040357 33808505 PMC8066904

[B24] Miyamoto S. Kollman P. A. (1992). Settle: an Analytical Version of the SHAKE and RATTLE Algorithm for Rigid Water Models. J. Comput. Chem. 13, 952–962. 10.1002/jcc.540130805

[B25] Mok N. Y. Brown N. (2017). Applications of Systematic Molecular Scaffold Enumeration to Enrich Structure-Activity Relationship Information. J. Chem. Inf. Model. 57 (1), 27–35. 10.1021/acs.jcim.6b00386 27990817 PMC6152611

[B26] Naghibzadeh S. (2001). castP: Computed Atlas of Surface Topography of Proteins. Biophysical J. 80 (1), 320a. 10.1366/0003702011953612 PMC168919 12824325

[B27] Nie Q. Du X. G. Geng M. Y. (2011). Small Molecule Inhibitors of Amyloid Beta Peptide Aggregation as a Potential Therapeutic Strategy for Alzheimer's Disease. Acta pharmacologica Sinica 32 (5), 545–551. 10.1038/aps.2011.14 21499284 PMC4002509

[B28] Rauk A. (2008). Why Is the Amyloid Beta Peptide of Alzheimer's Disease Neurotoxic. Dalton Trans. 10, 1273–1282. 10.1039/b718601k 18305836

[B29] Rishton G. M. LaBonte K. Williams A. J. Kassam K. Kolovanov E. (2006). Computational Approaches to the Prediction of Blood-Brain Barrier Permeability: A Comparative Analysis of central Nervous System Drugs versus Secretase Inhibitors for Alzheimer's Disease. Curr. Opin. Drug Discov. Development 9 (3), 303–313. 16729726

[B30] Shityakov S. Broscheit J. Förster C. (2012). Alpha-Cyclodextrin Dimer Complexes of Dopamine and Levodopa Derivatives to Assess Drug Delivery to the central Nervous System: ADME and Molecular Docking Studies. Int. J. nanomedicine 7, 3211–3219. 10.2147/ijn.s31373 22811606 PMC3394464

[B31] Shityakov S. Fischer A. Su K. P. Hussein A. A. Dandekar T. Broscheit J. (2020). Novel Approach for Characterizing Propofol Binding Affinities to Serum Albumins from Different Species. ACS Omega 5 (40), 25543–25551. 10.1021/acsomega.0c01295 33073080 PMC7557242

[B32] Shityakov S. Forster C. (2014). In Silico predictive Model to Determine Vector-Mediated Transport Properties for the Blood-Brain Barrier Choline Transporter. Adv. Appl. Bioinform Chem. 7, 23–36. 10.2147/aabc.s63749 25214795 PMC4159400

[B33] Shityakov S. Hayashi K. Störk S. Scheper V. Lenarz T. Förster C. Y. (2021). The Conspicuous Link between Ear, Brain and Heart–Could Neurotrophin-Treatment of Age-Related Hearing Loss Help Prevent Alzheimer’s Disease and Associated Amyloid Cardiomyopathy. Biomolecules 11 (6), 900. 10.3390/biom11060900 34204299 PMC8235707

[B34] Shityakov S. Roewer N. Förster C. Broscheit J.-A. (2017). In Silico investigation of Propofol Binding Sites in Human Serum Albumin Using Explicit and Implicit Solvation Models. Comput. Biol. Chem. 70, 191–197. 10.1016/j.compbiolchem.2017.06.004 28917201

[B35] Shityakov S. Salvador E. Pastorin G. Förster C. (2015). Blood-brain Barrier Transport Studies, Aggregation, and Molecular Dynamics Simulation of Multiwalled Carbon Nanotube Functionalized with Fluorescein Isothiocyanate. Int. J. Nanomedicine 10, 1703–1713. 10.2147/ijn.s68429 25784800 PMC4356663

[B36] Shityakov S. Sohajda T. Puskás I. Roewer N. Förste C. Broscheit J. A. (2014). Ionization States, Cellular Toxicity and Molecular Modeling Studies of Midazolam Complexed with Trimethyl-Beta-Cyclodextrin. Molecules 19 (10), 16861–16876. 10.3390/molecules191016861 25338177 PMC6270744

[B37] Takahashi R. H. Nagao T. Gouras G. K. (2017). Plaque Formation and the Intraneuronal Accumulation of Beta-Amyloid in Alzheimer's Disease. Pathol. Int. 67 (4), 185–193. 10.1111/pin.12520 28261941

[B38] Vainio M. J. Kogej T. Raubacher F. Sadowski J. (2013). Scaffold Hopping by Fragment Replacement. J. Chem. Inf. Model. 53 (7), 1825–1835. 10.1021/ci4001019 23826858

[B39] Voicu A. Duteanu N. Voicu M. Vlad D. Dumitrascu V. (2020). The Rcdk and Cluster R Packages Applied to Drug Candidate Selection. J. cheminformatics 12 (1), 3. 10.1186/s13321-019-0405-0 PMC6970292 33430987

[B40] Wallace A. C. Laskowski R. A. Thornton J. M. (1995). Ligplot - a Program to Generate Schematic Diagrams of Protein Ligand Interactions. Protein Eng. 8 (2), 127–134. 10.1093/protein/8.2.127 7630882

[B41] Walti M. A. Ravotti F. Arai H. Glabe C. G. Wall J. S. Böckmann A. (2016). Atomic-resolution Structure of a Disease-Relevant Abeta(1-42) Amyloid Fibril. Proc. Natl. Acad. Sci. United States America 113 (34), E4976–E4984. 10.1073/pnas.1600749113 PMC5003276 27469165

[B42] Wang W. W. Xu P. Tang H. (2015). Sustainable Production of Valuable Compound 3-Succinoyl-Pyridine by Genetically Engineering Pseudomonas Putida Using the Tobacco Waste. Scientific Rep. 5. 10.1038/srep16411 PMC4647180 26574178

[B43] Zhang Y. Zhao Y. Wang Z. Gong H. Ma L. Sun D. (2018). Menadione Sodium Bisulfite Inhibits the Toxic Aggregation of Amyloid-Beta(1-42). Biochim. Biophys. Acta Gen. subjects 1862 (10), 2226–2235. 10.1016/j.bbagen.2018.07.019 30036601

