# Supplementary figures and images for: Scaffold Searching of FDA and EMA-Approved Drugs Identifies Lead Candidates for Drug Repurposing in Alzheimer’s Disease
Source: Front Chem. 2021 Oct 22;9:736509. doi: 10.3389/fchem.2021.736509 (PMC8571023; doi:10.3389/fchem.2021.736509)

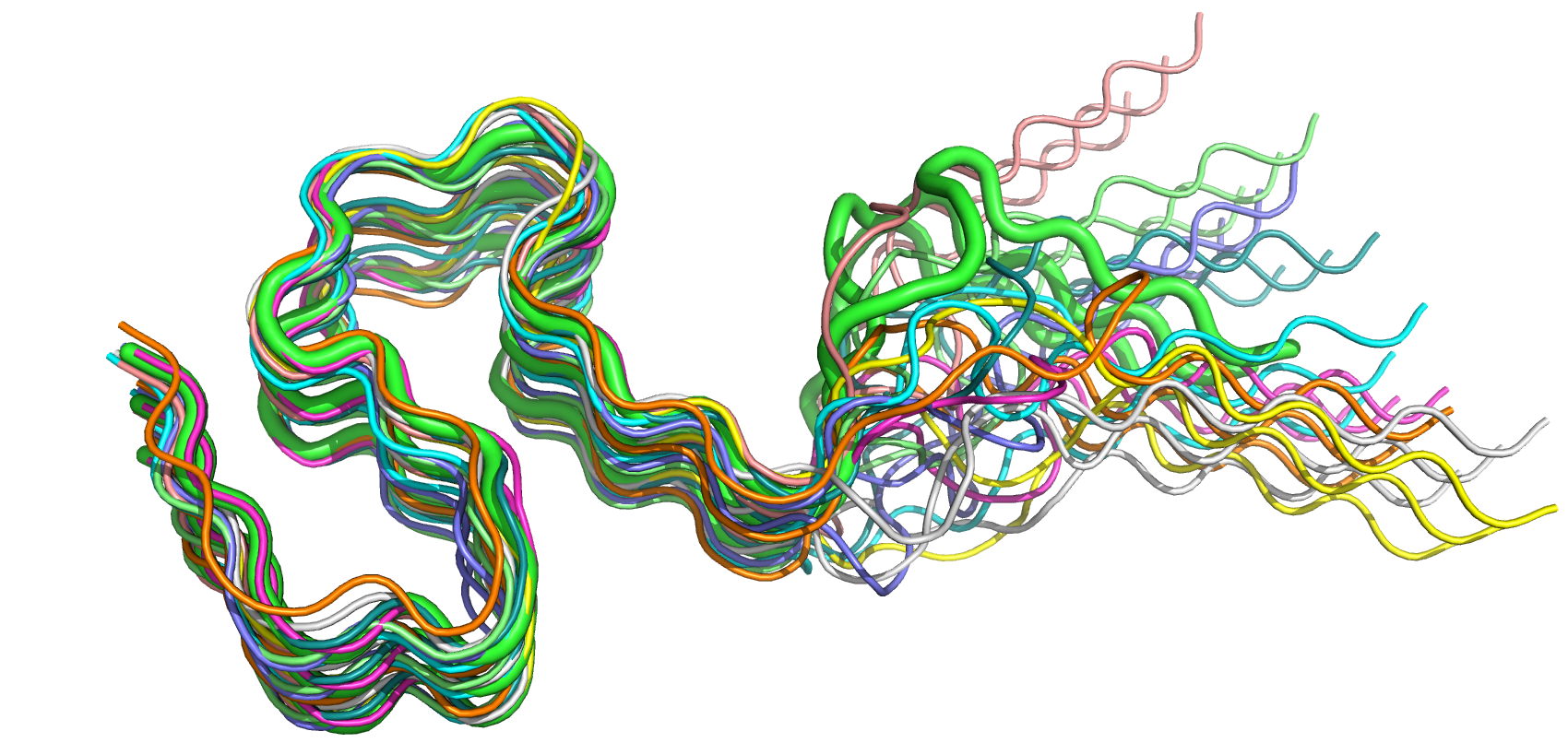

Supplement: Supplementary file 2 [file DataSheet2.zip › Supplementary data/conformers/pics/pic1.png]

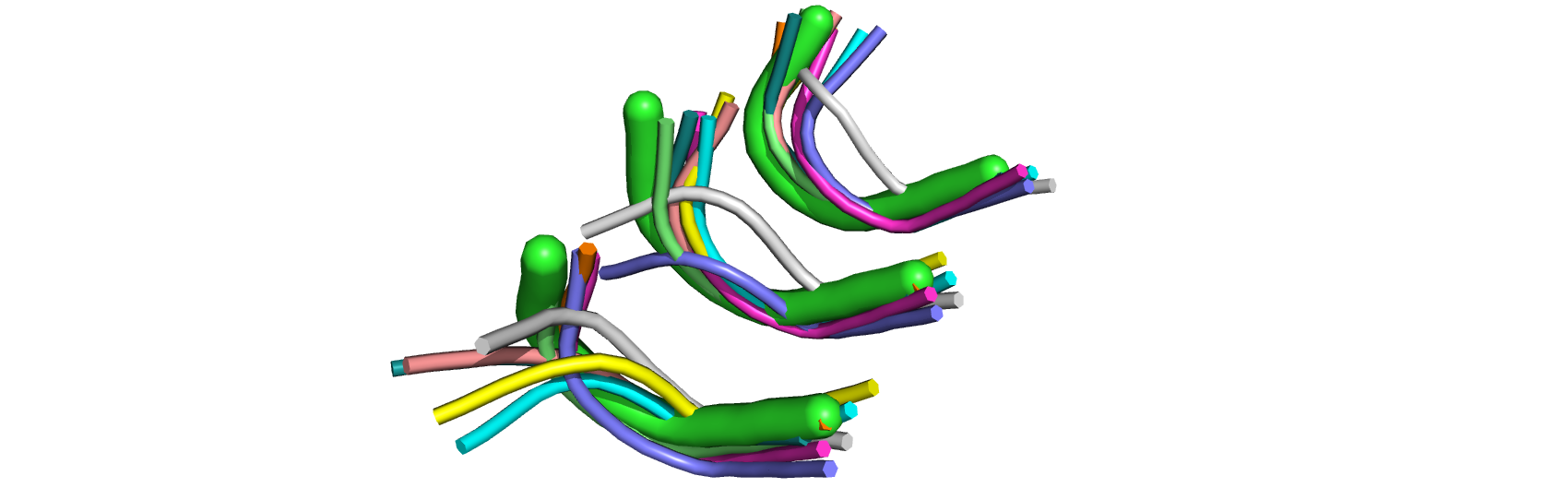

Supplement: Supplementary file 2 [file DataSheet2.zip › Supplementary data/conformers/pics/pic2.png]
